# Between-hospital variation in biopsy indication for patients with newly diagnosed glioblastoma in the Dutch Quality Registry for Neurosurgery

**DOI:** 10.1007/s11060-025-04959-5

**Published:** 2025-02-06

**Authors:** Ilaria Viozzi, Gerjon Hannink, Hilko Ardon, Rutger K. Balvers, Lisette Bosscher, Sarita van Geest, Vincent K. Y. Ho, Koos Hovinga, Lesley Kwee, Rishi Nandoe Tewarie, Pierre A. Robe, Olivier van der Veer, Michiel Wagemakers, Mark ter Laan, Philip C. De Witt Hamer

**Affiliations:** 1 Department of Neurosurgery, Radboud University Medical Center, Radboud Institute for Health Sciences, 6525 GA Nijmegen, The Netherlands; 2 Department of Medical Imaging, Radboud University Nijmegen Medical Centre, Nijmegen, The Netherlands; 3 Department of Neurosurgery, Elisabeth-Tweesteden Hospital, Tilburg, The Netherlands; 4 Department of Neurosurgery, Brain Tumour Centre, Erasmus University Medical Centre, Rotterdam, The Netherlands; 5 Department of Neurosurgery Isala Hospital, Zwolle, The Netherlands; 6 Department of Neurosurgery, Martini Hospital, Groningen, The Netherlands; 7 Department of Research & Development, Netherlands Comprehensive Cancer Organisation (IKNL), Utrecht, The Netherlands; 8 Department of Neurosurgery, Maastricht University Medical Centre, Maastricht, The Netherlands; 9 Department of Neurosurgery, Northwest Clinics, Alkmaar, The Netherlands; 10 Department of Neurosurgery, Haaglanden Medical Centre, The Hague, The Netherlands; 11 Department of Neurosurgery, University Medical Centre Utrecht, Utrecht, The Netherlands; 12 Department of Neurosurgery, Medical Spectrum Twente, Enschede, The Netherlands; 13 Department of Neurosurgery, University Medical Centre Groningen, Groningen, The Netherlands; 14 Department of Neurosurgery, Amsterdam University Medical Centre, Amsterdam, The Netherlands

**Keywords:** Glioblastoma, Variation, Practice, Biopsy, Resection

## Abstract

**Purpose:**

This registry-based study aims to examine the variation in biopsy and resection indications for glioblastoma patients across Dutch hospitals and to identify patient- and hospital-related factors associated with the surgical treatment.

**Methods:**

Data from all 7443 adults with first-time glioblastoma surgery at 12 hospitals were obtained from the prospective population-based Quality Registry Neurosurgery in the Netherlands between 2011 and 2021. Patients were stratified by either biopsy or resection. We analyzed variation in American Association of Anesthesiologist (ASA) classification, Karnofsky Performance Score (KPS), gender and age distribution between the different centers. Between-hospital variation in biopsy percentage was analyzed using a funnel plot. Logistic regression was used to identify associated patient- and hospital-related factors.

**Results:**

In total, 32% of the newly diagnosed glioblastoma patients underwent a biopsy, with wide variations between the different centers (23–56%). Patients-related variables such as higher age or ASA classification and lower KPS were significantly associated with the indication for biopsy. After correction for these factors, between-hospital variation persisted, with two institutes performing more biopsies than expected and one less than expected. Median overall survival was 12.5 months (95% CI 12.2–12.9) in the resection group and 5.6 months (95% CI 5.1–6) in the biopsy group, with wide variations between the different centers.

**Conclusion:**

A substantial between-hospital variation in biopsy percentages was found. Patient factors (age, ASA classification and KPS) but also hospital factors (such as academic setting) impact surgical decisions. Variation persisted also after correction for potential confounders, indicating that other factors play a role in decision-making.

**Supplementary Information:**

The online version contains supplementary material available at 10.1007/s11060-025-04959-5.

## Introduction

The standard treatment for patients with glioblastoma consists of maximal safe resection followed by adjuvant concurrent chemo-radiotherapy [1]. Cytoreductive surgical resection is the treatment of choice in most glioblastoma cases, as it has been shown to improve survival in combination with chemoradiation [2, 3]. Risk of morbidity and mortality is mostly determined by the tumour location, size, and the patient’s comorbidities. When a resection is not deemed safe, a stereotactic or open biopsy is necessary to confirm the radiologically suspected diagnosis before starting the adjuvant treatment [1]. Patients presenting with a poor clinical condition at the onset of their disease, often characterized by a Karnofsky Performance Score (KPS) lower than 70 which is deemed unlikely to improve after reduction of mass effect, typically receive best supportive care (BSC) without histopathological verification [4, 5].

Several studies indicate that patients undergoing a biopsy have significant shorter survival than patients undergoing surgical resection [6–10]. However, it remains difficult to establish whether patients undergoing a biopsy face a shorter survival because they miss the advantage of a resection or if patients selected for biopsy inherently have a less favorable course of the disease, because of multifocality, deep-seated location or poorer condition upon presentation.

The decision to recommend patients with glioblastoma either a biopsy or a resection is inherently complex. It relies on a thorough evaluation by multidisciplinary boards, treating physicians, and the patients themselves [1]. This process integrates a wide range of medical information, including the patient’s overall health, tumour characteristics, and potential treatment outcomes. Additionally, patient preferences play a crucial role, ensuring that their values and wishes are respected. This collaborative approach ensures that all relevant factors are considered, leading to a well-informed and personalized treatment plan. In a previously published study on glioblastoma surgery outcomes in the Dutch Quality Registry for Neurosurgery (QRNS), longer overall survival (OS) was associated with patient-related factors (lower age, better clinical performance)[11]. Biopsy percentages varied widely in this study, which is consistent with other literature reports [2], and longer overall survival was also associated with a lower biopsy percentage. This registry-based study was designed to examine the variation in indications for biopsy or resection for patients with newly diagnosed glioblastoma across hospitals in the Netherlands, to identify patient- and hospital-related factors associated with the decision between biopsy and resection and explored how these variations impact patient survival.

## Methods

We performed a nationwide registry-based cohort study performed in the Netherlands. Data were prospectively registered in the Quality Registry Neurosurgery (QRNS). This registry provided feedback to all institutions with neurosurgical departments on patient outcomes and treatment variation for self-assessment and for monitoring of quality of care. Participation in the registry was mandatory for all institutions providing glioblastoma surgery. A de-identified data set for analysis was provided by an independent third party (Healthcare Information Provision Foundation [12]).

## Patients

All adult patients (age ≥ 18 years old) who underwent first-time glioblastoma surgery in the Netherlands between January 2011 and December 2021 were included in the study. Patients were included if the diagnosis glioblastoma was histologically confirmed, according to the 2007, 2016 or 2021 WHO Classification of Tumours of the Central Nervous System [13–15]. Each patient received treatment at one of twelve Dutch neurosurgical centers providing neuro-oncological care. Patients underwent either a gross-total or partial surgical resection or biopsy, defined as surgical removal of tissue for diagnosis only, either by open or needle biopsy. The registry does not include patients who did not have surgery (resection nor biopsy) for a radiologically suspected glioblastoma and instead received best supportive care.

## Data collection

Data was prospectively collected and registered by neurosurgeons, nurse specialists in neuro-oncology or trained physicians. Treatment decisions for patients were made in multidisciplinary tumour board meetings in all hospitals.

Collected data included age at diagnosis, gender, Karnofsky Performance Score (KPS) before surgery, American Society of Anesthesiologists (ASA) classification before surgery, the chosen adjuvant treatment (combined chemotherapy and radiotherapy, radiotherapy only, chemotherapy only, best supportive care), date of death.

The dates of death were verified and updated against the information available from the Netherlands Cancer Registry (NCR). The NCR collects information on all newly diagnosed cancer patients in the Netherlands following notification by the pathology laboratories. Information on vital status was retrieved through yearly linkage with the Municipal Personal Records Database.

The QRNS database comprises data for the quality of care evaluation in accordance with the Dutch Quality Act for Healthcare, therefore written informed consent was not needed [16]. Ethical approval was not necessary because the study is not subject to the Medical Research Involving Human Subjects Act (WMO) [17]. After delivery by a trusted third party [12], de-identified data was analyzed. Three authors (IV, GH, PWH) had full access to the data and were responsible for the analysis.

## Outcomes

Our aim was to assess practice variation in surgical decision making by examining biopsy and resection percentages. We evaluated these percentages annually nationwide and for each individual center over the entire study period. Additionally, we analyzed the distribution of KPS and ASA classification within the biopsy and resection groups, both at the population level and within each center.

To further explore the practice variation, we report overall survival (OS) in patients who underwent biopsy versus those who underwent resection for both the overall population and for the different centers, including the variation in the adjuvant treatment.

## Statistical analysis

For the presentation of baseline data, descriptive statistics were used. Continuous variables are depicted as mean ± standard deviation (SD), categorical variables as frequencies or percentages. The biopsy and resection groups were considered inherently distinct; thus, no comparative analysis was conducted between them. A descriptive analysis was used to better characterize the two populations.

Variation in biopsy percentage was compared between centers using funnel plots. Funnel plots were created using the expected number of events as a measure of precision, and the risk-standardized observed-to-expected event ratios per hospital as indicators, as previously described [18]. To account for patients’ variation, the funnel plots were adjusted for age, KPS and ASA classification at diagnosis. Academic and non-academic setting of the different hospitals are specified in the plot. The funnel control limits, which help identify outliers, were derived from the Poisson distribution as 95 and 99% prediction limits. Univariable and multivariable regression analyses were performed to identify association between biopsy percentage and factors possibly influencing the surgical choice. We examined patient-related variables, i.e., age, sex, ASA classification, and KPS. Hospital-related variables included the type of facility (academic or non-academic) and case load, determined by the total patient volume per center.

Survival was analyzed with censoring at the last date of follow-up or the lookup date of alive status, and analyses were based on complete cases. The Kaplan–Meier curves were used to show overall survival for the entire population and per center, showing the variation across the several institutes.

Statistical analyses were performed using R (version 4.3.3, R Foundation for Statistical Computing, Vienna, Austria).

## Results

Between January 2011 and December 2021, 7443 patients were diagnosed with a glioblastoma in the Netherlands and were registered in the QRNS. Of these, 2362 (32%) underwent a biopsy and 5081 (68%) underwent a resection. Biopsy percentage showed limited variability over the years (see supplementary information) varying between a minimum of 26% (2016) and a maximum of 36% (in 2013).

**Fig. 1:**
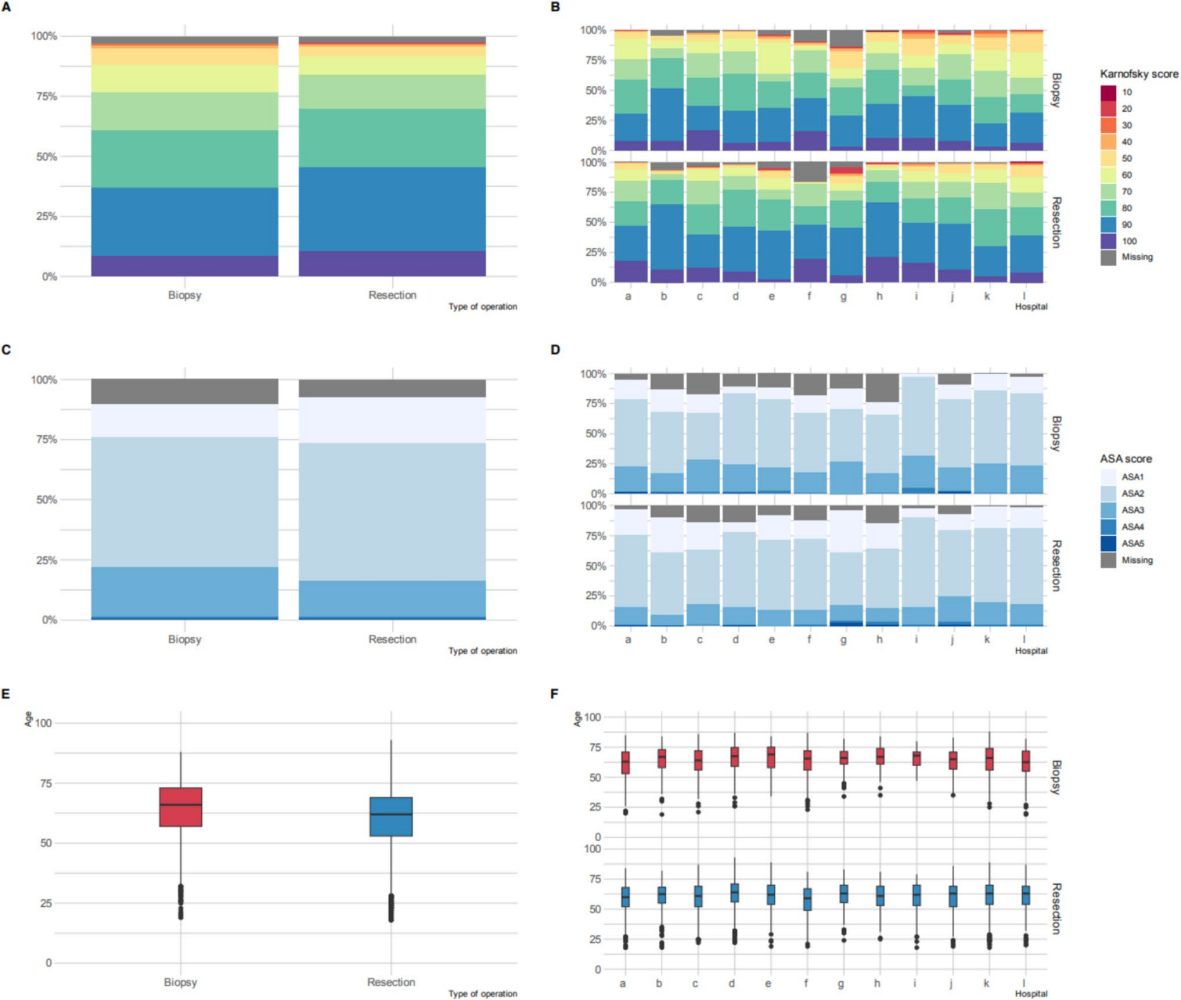
Pre-operative KPS, ASA classification, and age distribution in biopsy and resection groups. Plots on the left-side show results for the entire population, stratified by type of operation. Plots on the right-side show results per center, stratified by type of operation

Mean age at surgery was 64 (± 11) years in the biopsy group and 61 (± 12) years in the resection group. Gender distribution was 59% male in the biopsy group and 60% male in the resection group. Patients in the resection group more frequently showed a pre-operative KPS above 70 (86%) than patients in the biopsy group (79%), as well as more frequently an ASA classification 1 or 2 (82% versus 75%). Figure 1 illustrates the overall frequency of KPS, ASA classification, and age in both the biopsy and resection groups. The data is presented for the entire groups and is further divided by hospital and type of operation, highlighting the variations across different hospitals. The supplemental table comprises an overview of patient characteristics in both groups, listed per center, including missing data.

**Fig. 2:**
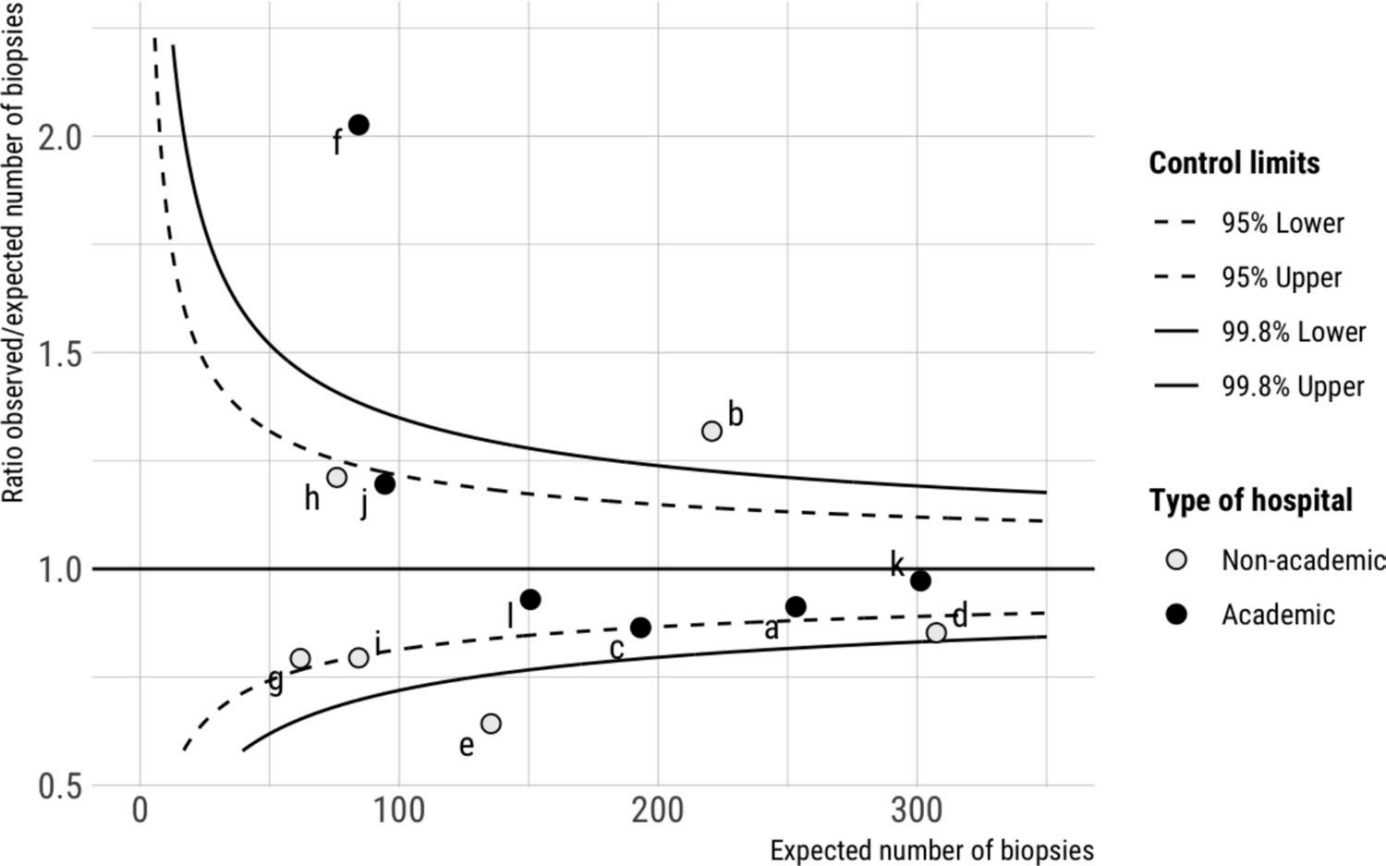
Funnel plots for expected biopsy. Each dot represents an institute indicated by a letter. The funnel plot is adjusted for ASA classification, KPS, setting, sex and age at diagnosis. Setting (academic and non-academic) is highlighted with colors. Ratios higher than 1.0 indicate a higher number of observed biopsies than expected

The biopsy percentages varied in the Dutch hospitals from a minimum of 23% to a maximum of 56%. The between-institutions variation in biopsy percentage is displayed in a funnel plot (Fig. 2). The funnel plot was adjusted according to pre-operative ASA classification, KPS and age to account for patients’ variation. Ratios higher than 1.0 indicate a higher biopsy percentage than expected based on the standardized ratio. In institute e the number of observed biopsies was less than expected (23%). In institutes b and f more biopsies were performed than expected (respectively 43% and 56%). Other institutions had ratios within the control limits, i.e., observed events were according to expectations.

Table 1 reports the univariable and multivariable linear regression analysis for association with indication biopsy. In the multivariable analyses, performed on 6379 completed cases, higher age, ASA 3, lower KPS and non-academic setting were statistically significantly associated with indication for biopsy.

**Table 1:** Univariable and multivariable logistic regression analysis for association with indication biopsy

	Univariable		Multivariable	
	OR (95% C.I.)	p-value	OR (95% C.I.)	p-value
Age	1.03 (1.02–1.03)	0	1.02 (1.02–1.03)	** < 0.001**
Female vs Male gender	1.01 (0.91–1.12)	0.887	0.94 (0.84–1.05)	0.247
ASA 2 vs ASA 1	1.30 (1.13–1.50)	** < 0.001**	1.10 (0.94–1.28)	0.239
ASA 3 vs ASA 1	1.89 (1.60–2.24)	** < 0.001**	1.38 (1.15–1.66)	** < 0.001**
ASA 4–5 vs ASA 1	1.49 (0.92–2.34)	0.093	1.05 (0.63–1.72)	0.836
KPS 70–80 vs KPS < 70	0.69 (0.60–0.80)	** < 0.001**	0.68 (0.58–0.79)	** < 0.001**
KPS 90–100 vs KPS < 70	0.55 (0.47–0.63)	** < 0.001**	0.60 (0.51–0.70)	** < 0.001**
Academic setting	0.73 (0.66–0.80)	** < 0.001**	0.75 (0.67–0.83)	** < 0.001**
Caseload	1.00 (1.00–1.00)	** < 0.001**	1.00 (1.00–1.00)	0.145

*OR* Odds Ratios, *C.*I. confidence intervals, *ASA* American Society of Anesthesiologists, *KPS* Karnofsky Performance Score

**Fig. 3:**
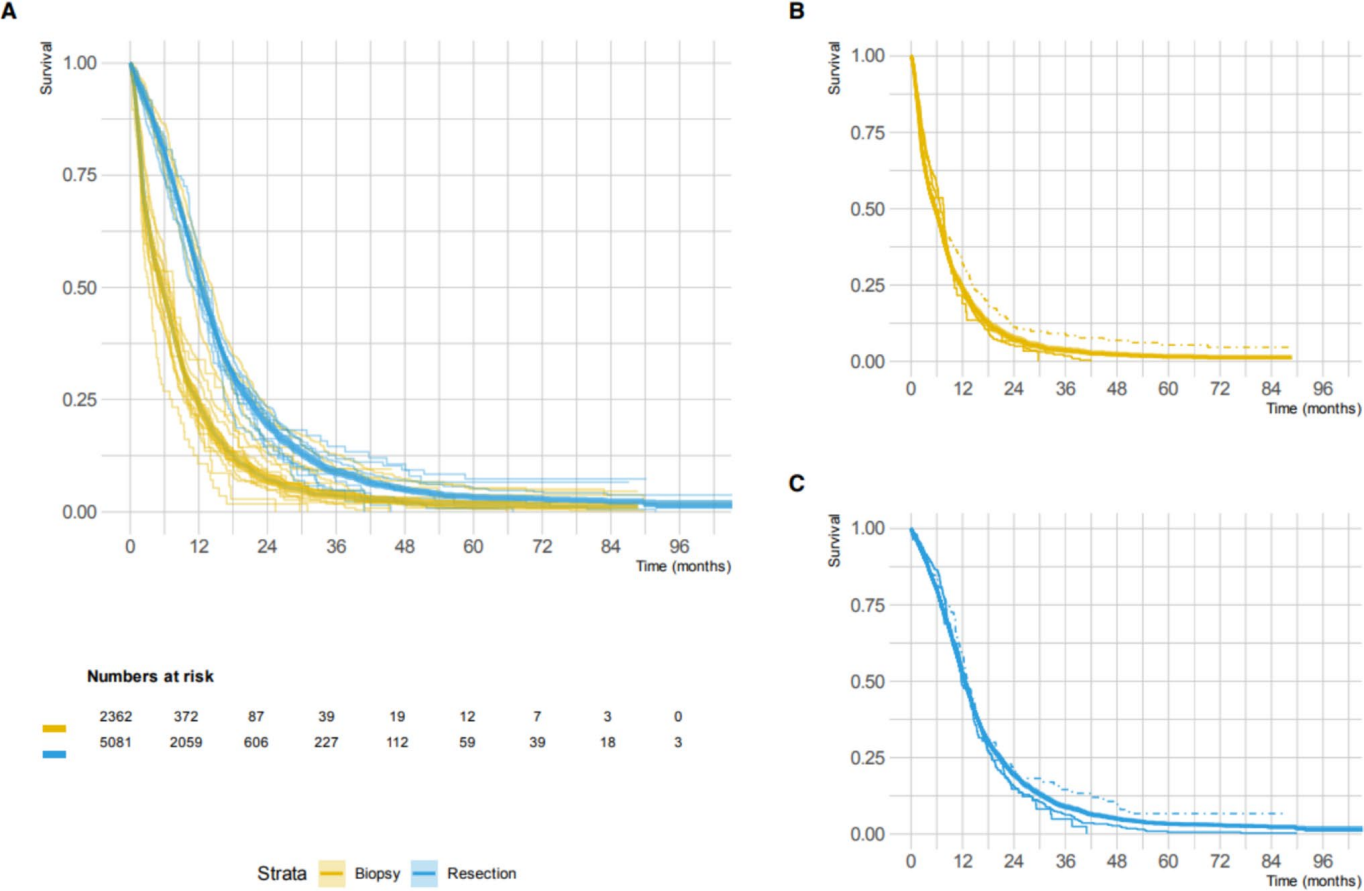
Kaplan–Meier survival curves. **A** Kaplan–Meier curves showing OS for biopsy (yellow) and resection (blue). Thick curves represent OS for the entire population with 95% confidence intervals. Thin curves represent OS per center. Numbers at risk refer to the entire populations. **B** and **C** Kaplan–Meier curves of the outliers centers for the biopsy group (**B**) and resection (**C**). Thick line represents OS, dashed line represents the center with less biopsy than expected and solid lines represent the centers with more biopsy than expected

Median overall survival was 12.5 months (95% CI 12.2–12.9) in the resection group and 5.6 months (95% CI 5.1–6) in the biopsy group. Median survival varied per center for both the biopsy group (range 3.3–7.6 months) and resection group (range 10.9–14.5 months). The median survival and survival curves calculated for the outliers from the funnel plot (b, f, e) were not substantially different from the median overall survival (for centers with higher biopsy percentages b and f 6.9 and 7.6 months in the biopsy groups and 12.8 and 13.1 months in the resection groups, respectively; for center e with lower biopsy percentages 6.7 months in the biopsy groups and 12.2 months in the resection group). Survival curves for the biopsy and resection group are depicted in Fig. 3.

**Fig. 4:**
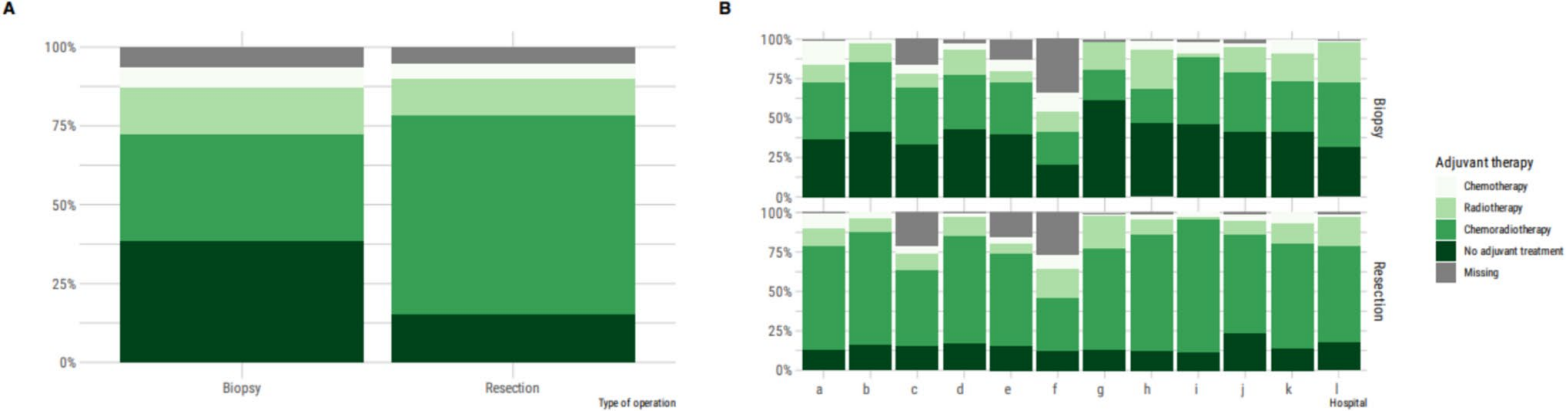
Distribution of post-operative treatment in the biopsy and resection group. **A** results for the entire population, stratified by type of operation. **B** results per center, stratified by type of operation

After surgical resection, post-operative treatment consisted of adjuvant combined chemotherapy and radiotherapy in 67%, only chemotherapy in 5% and only radiotherapy in 12%. After biopsy, post-operative treatment consisted of adjuvant combined chemotherapy and radiotherapy in 36%, only chemotherapy 7% and only radiotherapy in 15%. Best supportive care was chosen in 40% of patients after biopsy and 16% after resection (Fig. 4). The post-operative treatment after surgery varied among the different centres.

## Discussion

The main findings of the study are: (1) between 2011 and 2021 in the Netherlands 32% of patients with a newly diagnosed glioblastoma underwent a biopsy, with a wide variation among Dutch centers; (2) patient-related variables such as higher age or ASA classification and lower KPS were associated with the indication for biopsy; hospitals with an academic setting performed less biopsies than hospitals with a non-academic setting. (3) Survival ranges were variable for both the biopsy and resection groups. Survival of patients in the hospitals with higher and lower biopsy percentage was not substantially different from the median overall survival. Interestingly, in over 40% of the patients who received a biopsy, no adjuvant treatment was performed.

Our study revealed that approximately one-third of glioblastoma patients in the Netherlands are not deemed candidates for surgical resection, consistent with findings from other population-based studies [19, 20]. Studies indicate that biopsy rates vary widely, ranging from 3 to 44% [2, 19–24] influenced by multiple factors. Typically, biopsy rates are lower in randomized controlled trials (RCTs), likely due to the generally better clinical and neurological condition of RCT participants [2, 3, 21]. Additionally, evolving research, clinical guidelines, and geographic differences further contribute to this variability.

Our study focuses on the variation in biopsy rates for glioblastoma patients across hospitals in the same country, revealing significant practice differences. Both patient-related and hospital-related factors were associated with the choice of biopsy. Interestingly, despite accounting for these variables, the variation persisted, suggesting that additional factors, not considered in this analysis, contribute to the clinical variation. Tumour-related factors like volume, location, ependymal or callosal expansion are known to play a role in this decision [25]. Unfortunately, these factors could not be analyzed, as these are not provided in this national registry. Clinical variation may also be related to the local expertise and available resources, like 5-ALA use, neuromonitoring, and awake surgery. Geographical and cultural differences possibly also play a role, such as religion or quality of life perception. One crucial aspect to consider is that the decision between biopsy and resection involves a comprehensive evaluation of the patient’s unique situation, including tumour location and size and overall patient health. Additionally, patient preferences, prioritizing quality of life or seeking aggressive treatment, play a crucial role. These aspects, collectively evaluated within the multidisciplinary boards contribute to make informed decisions. Capturing these nuanced factors in data registries is challenging, since patient preferences, clinical judgment, and multidisciplinary discussions are difficult to quantify. As a result, some critical aspects influencing the decision remain unrecorded.

In our study, we observed wide ranges of survival for both biopsy and resection. The median OS observed in our series aligns with the OS reported in other studies [19, 20, 26]. Noteworthy, the survival rates in centers where a higher or lower percentage of biopsies were performed did not significantly differ from the rest. In a previously published paper with QRNS data, a lower survival was associated with higher biopsy rates [27]. It is important to underline that patients’ selection likely impact these results, as hospitals may have different criteria for recommending biopsies. For instance, in patients with a radiologically suspected glioblastoma in a poor clinical condition, older age, and a low KPS, some hospitals might opt for best supportive care without histological diagnosis. Hence, patients with the worse clinical condition and prognosis were not included in the analysis, impacting both biopsy percentage and survival. This may also result in a (significant?) variation between centers that cannot be corrected for.

Another interesting finding of our study is the proportion of patients who did not receive adjuvant treatment after surgery. Specifically, 40% of patients who underwent a biopsy did not receive adjuvant treatment. A possible explanation is that a biopsy might be performed to confirm the suspected diagnosis, even if the patient’s clinical condition is not optimal for starting oncological treatment. Additionally, disease progression may occur after a biopsy, as it does not lead to mass reduction. This finding highlights the importance of considering individual patient factors and tailoring treatment decisions and raises the question whether performing a biopsy should be advised in patients with a compromised neurological condition at disease presentation. Remarkably, we noticed substantial variation also in the post-operative treatment choices. This observation underscores the critical influence of factors such as patient preferences, clinical judgment, and multidisciplinary discussions in determining the selected treatment approach.

The major strength of this study is that it includes all Dutch neurosurgical centers, reflecting the current treatment landscape in the country and implementing discussions and improvements on quality of glioblastoma care in the Netherlands. Results from this study can be used to inform patients using real-world data, more reliable and generalizable than results from RCTs. As limitation, the data set was deliberately restricted to essential items to decrease the administrative burden of the registry. Consequently, we lack some information on clinical, radiological, and histological characteristics and centers-specific details. Regarding the survival exploration, known prognostic factors such as tumour locations and volume, molecular classifications, and other therapies could not be included in this analysis. Future studies should include molecular data as well, fundamental especially when analyzing survival. We did not measure the health-related quality of life, which represent a fundamental aspect of decision making in glioblastoma care. Another limitation of our study is the potential impact of missing data, which may affect the accuracy and generalizability of our findings. Future studies could also differentiate decisions in multidisciplinary tumor boards by conference composition and center certification.

## Conclusion

In conclusion, our study revealed that almost one third of the patients diagnosed with a newly diagnosed glioblastoma is not deemed suitable for surgical resection. Substantial practice variation exists among Dutch centers in biopsy percentages. Both patient-related, particularly higher age and ASA classification and lower KPS, and hospital-related factors, particularly non-academic setting, were associated with the choice of biopsy. Interestingly, despite accounting for these variables, the variation persisted, suggesting that additional factors, not considered in this analysis, contribute to the clinical variation.

These findings underscore the critical role of clinical registries in identifying key differences in clinical practices and healthcare delivery, as well as their impact on patient outcomes and access to adjuvant treatments. This study significantly contributes to our understanding of the decision-making process and the standardization of care, and the enhancement of quality.

## Supplementary Information

Below is the link to the electronic supplementary material.

Supplementary file1 (PDF 17 KB)

Supplementary file2 (DOCX 48 KB)

## Data Availability

The data (deidentified patients data) that support the findings of this study are available on request from the corresponding author after publication, after providing a methodologically sound proposal.
