# Supplementary figures and images for: Between-hospital variation in biopsy indication for patients with newly diagnosed glioblastoma in the Dutch Quality Registry for Neurosurgery
Source: J Neurooncol. 2025 Feb 6;172(3):625–32. doi: 10.1007/s11060-025-04959-5 (PMC11968504; doi:10.1007/s11060-025-04959-5)

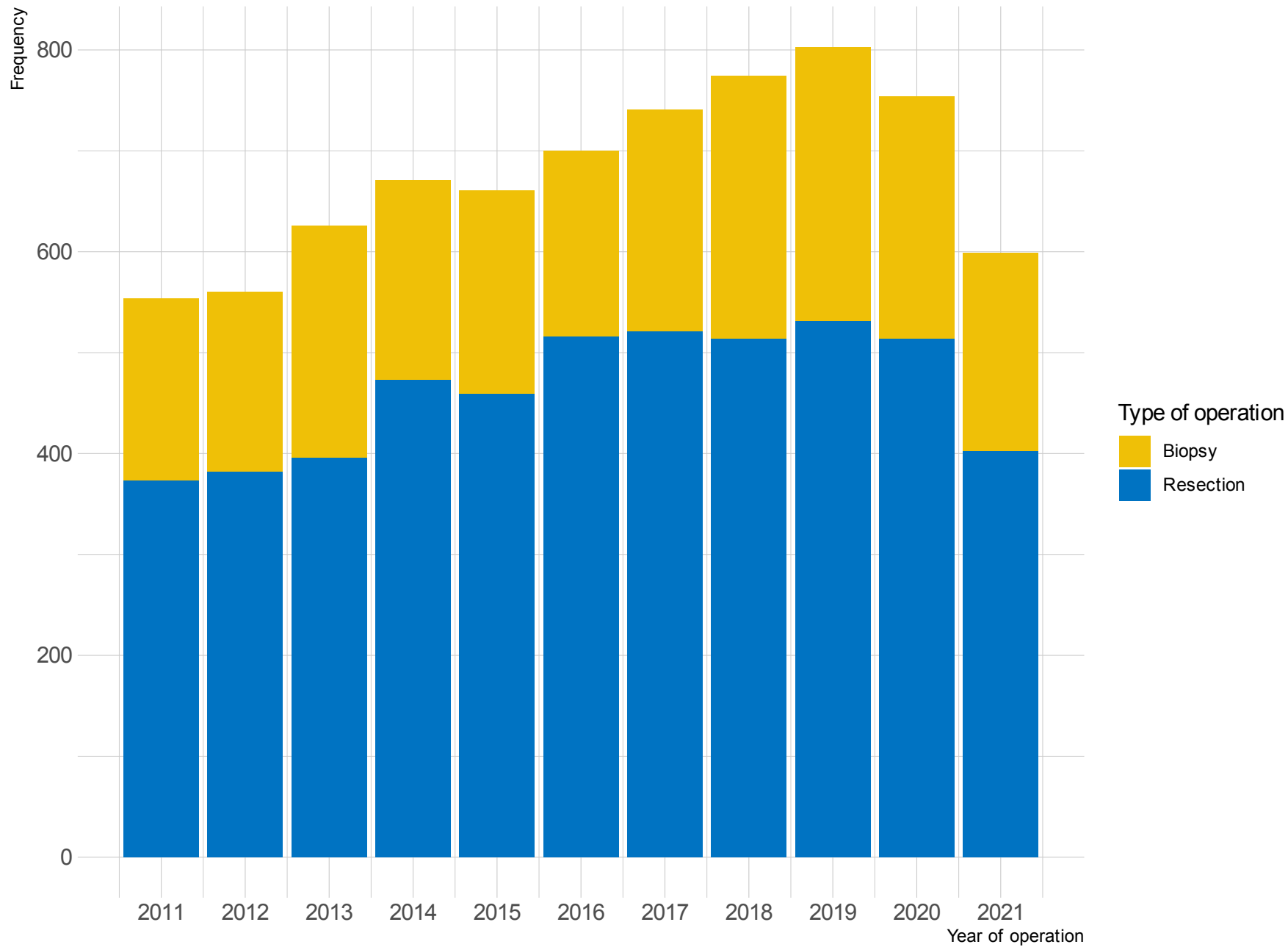

Supplement: Supplementary file 1 — Supplementary file1 (PDF 17 KB) [file 11060_2025_4959_MOESM1_ESM.pdf]
