# Supplementary material for: Between-hospital variation in biopsy indication for patients with newly diagnosed glioblastoma in the Dutch Quality Registry for Neurosurgery
Source: J Neurooncol. 2025 Feb 6;172(3):625–32. doi: 10.1007/s11060-025-04959-5 (PMC11968504; doi:10.1007/s11060-025-04959-5)
Supplement: Supplementary file 2 — Supplementary file2 (DOCX 48 KB) [file 11060_2025_4959_MOESM2_ESM.docx]

|  | **Overall** | | **a** | | **b** | | **c** | | **d** | | **e** | | **f** | | **g** | | **h** | | **i** | | **j** | | **k** | | **l** | |
| --- | --- | --- | --- | --- | --- | --- | --- | --- | --- | --- | --- | --- | --- | --- | --- | --- | --- | --- | --- | --- | --- | --- | --- | --- | --- | --- |
|  | **Biopsy** | **Resection** | **Biopsy** | **Resection** | **Biopsy** | **Resection** | **Biopsy** | **Resection** | **Biopsy** | **Resection** | **Biopsy** | **Resection** | **Biopsy** | **Resection** | **Biopsy** | **Resection** | **Biopsy** | **Resection** | **Biopsy** | **Resection** | **Biopsy** | **Resection** | **Biopsy** | **Resection** | **Biopsy** | **Resection** |
| **N** | 2362 | 5081 | 258 | 749 | 416 | 546 | 209 | 637 | 296 | 737 | 104 | 348 | 248 | 190 | 67 | 139 | 125 | 164 | 67 | 183 | 128 | 239 | 297 | 742 | 147 | 407 |
| **Male (%)** | 1395 (59.1) | 3037  (59.8) | 160 (62.0) | 427 (57.0) | 216 (51.9) | 310 (56.8) | 141 (67.5) | 390 (61.2) | 167 (56.4) | 451 (61.2) | 63 (60.6) | 221 (63.5) | 136 (54.8) | 102 (53.7) | 35 (52.2) | 81  (58.3) | 76 (60.8) | 96  (58.5) | 43 (64.2) | 103 (56.3) | 82 (64.1) | 145 (60.7) | 182 (61.3) | 465 (62.7) | 94 (63.9) | 246 (60.4) |
| **Missing (%)** | 110 (4.7) | 192 (3.8) | 13  (5.0) | 44 (5.9) | 61 (14.7) | 82 (15.0) | 4 (1.9) | 8 (1.3) | 0 (0.0) | 1 (0.1) | 2 (1.9) | 1 (0.3) | 14 (5.6) | 18 (9.5) | 10 (14.9) | 8 (5.8) | 2 (1.6) | 6 (3.7) | 0 (0.0) | 0 (0.0) | 1 (0.8) | 1 (0.4) | 0 (0.0) | 3 (0.4) | 3 (2.0) | 20 (4.9) |
| **Age mean (SD)**  **Missing (%)** | 64.08 (11.5)  46 (1.9) | 60.51 (12.1)  72 (1.4) | 61.17 (13.4)  3(1.2) | 58.64 (12.41)  12 (1.6) | 65.04 (10.24)  28 (6.7) | 60.87 (10.80)  34 (6.2) | 63.08 (11.64)  1 (0,5) | 59.60 (12.25)  3 (0.5) | 65.95 (11.02)  2 (0.7) | 62.56 (11.81)  8 (1) | 66.54 (11.32)  1 (1) | 61.24 (12.00)  5 (1.4) | 63.32 (11.79)  6 (2.4) | 56.64 (14.00)  2 (1) | 64.97 (10.03)  0 | 61.73 (11.26)  0 | 66.29 (9.37)  2 (1.6) | 60.20 (11.52)  0 | 64.76 (8.96)  0 | 59.80 (12.68)  0 | 62.91 (10.75)  0 | 60.52 (12.26)  0 | 64.46 (11.95)  2 (0.7) | 61.39 (12.26)  5 0.7) | 61.54 (12.39)  1 (0.7) | 60.80 (11.70)  2 (0.5) |
| **KPS 10 (%)** | 2 (0.1) | 7 (0.1) | 0 (0.0) | 0 (0.0) | 0 (0.0) | 1 (0.2) | 0 (0.0) | 2 (0.3) | 0 (0.0) | 1 (0.1) | 0 (0.0) | 0 (0.0) | 1 (0.4) | 0 (0.0) | 0 (0.0) | 0 (0.0) | 1 (0.8) | 1 (0.6) | 0 (0.0) | 0 (0.0) | 0 (0.0) | 0 (0.0) | 0 (0.0) | 1 (0.1) | 0 (0.0) | 1 (0.2) |
| **KPS 20 (%)** | 11 (0.5) | 33 (0.6) | 1 (0.4) | 0 (0.0) | 0 (0.0) | 1 (0.2) | 0 (0.0) | 6 (0.9) | 1 (0.3) | 2 (0.3) | 1 (1.0) | 6 (1.7) | 1 (0.4) | 0 (0.0) | 1 (1.5) | 7 (5.0) | 0 (0.0) | 2 (1.2) | 1 (1.5) | 0 (0.0) | 2 (1.6) | 2 (0.8) | 2 (0.7) | 4 (0.5) | 1 (0.7) | 3 (0.7) |
| **KPS 30 (%)** | 11 (0.5) | 23 (0.5) | 0 (0.0) | 3 (0.4) | 0 (0.0) | 3 (0.5) | 1 (0.5) | 4 (0.6) | 0 (0.0) | 2 (0.3) | 1 (1.0) | 1 (0.3) | 1 (0.4) | 1 (0.5) | 0 (0.0) | 1 (0.7) | 0 (0.0) | 0 (0.0) | 1 (1.5) | 2 (1.1) | 0 (0.0) | 0 (0.0) | 6 (2.0) | 1 (0.1) | 1 (0.7) | 5 (1.2) |
| **KPS 40 (%)** | 33 (1.4) | 37 (0.7) | 3 (1.2) | 4 (0.5) | 1 (0.2) | 1 (0.2) | 3 (1.4) | 4 (0.6) | 2 (0.7) | 6 (0.8) | 1 (1.0) | 3 (0.9) | 3 (1.2) | 0 (0.0) | 2 (3.0) | 2 (1.4) | 1 (0.8) | 0 (0.0) | 3 (4.5) | 4 (2.2) | 1 (0.8) | 1 (0.4) | 10 (3.4) | 8 (1.1) | 3 (2.0) | 4 (1.0) |
| **KPS 50 (%)** | 158 (6.7) | 200 (3.9) | 15 (5.8) | 40 (5.3) | 17 (4.1) | 8 (1.5) | 13 (6.2) | 13 (2.0) | 18 (6.1) | 14 (1.9) | 4 (3.8) | 18 (5.2) | 2 (0.8) | 1 (0.5) | 9 (13.4) | 9 (6.5) | 9 (7.2) | 5 (3.0) | 9 (13.4) | 7 (3.8) | 9 (7.0) | 19 (7.9) | 31 (10.4) | 28 (3.8) | 22 (15.0) | 38 (9.3) |
| **KPS 60 (%)** | 271 (11.5) | 402 (7.9) | 44 (17.1) | 68 (9.1) | 24 (5.8) | 8 (1.5) | 19 (9.1) | 57 (8.9) | 30 (10.1) | 53 (7.2) | 27 (26.0) | 34 (9.8) | 10 (4.0) | 2 (1.1) | 6 (9.0) | 8 (5.8) | 12 (9.6) | 4 (2.4) | 7 (10.4) | 17 (9.3) | 11 (8.6) | 16 (6.7) | 50 (16.8) | 84 (11.3) | 31 (21.1) | 51 (12.5) |
| **KPS 70 (%)** | 375 (15.9) | 714 (14.1) | 42 (16.3) | 126 (16.8) | 36 (8.7) | 26 (4.8) | 43 (20.6) | 119 (18.7) | 56 (18.9) | 83 (11.3) | 7 (6.7) | 28 (8.0) | 46 (18.5) | 35 (18.4) | 5 (7.5) | 12 (8.6) | 17 (13.6) | 15 (9.1) | 10 (14.9) | 24 (13.1) | 27 (21.1) | 31 (13.0) | 66 (22.2) | 166 (22.4) | 20 (13.6) | 49 (12.0) |
| **KPS 80 (%)** | 568 (24.0) | 1247 (24.5) | 75 (29.1) | 151 (20.2) | 103 (24.8) | 112 (20.5) | 49 (23.4) | 163 (25.6) | 92 (31.1) | 229 (31.1) | 22 (21.2) | 91 (26.1) | 54 (21.8) | 30 (15.8) | 16 (23.9) | 31 (22.3) | 36 (28.8) | 28 (17.1) | 6 (9.0) | 37 (20.2) | 27 (21.1) | 53 (22.2) | 65 (21.9) | 223 (30.1) | 23 (15.6) | 99 (24.3) |
| **KPS 90 (%)** | 668 (28.3) | 1761 (34.7) | 59 (22.9) | 223 (29.8) | 184 (44.2) | 294 (53.8) | 42 (20.1) | 174 (27.3) | 78 (26.4) | 276 (37.4) | 30 (28.8) | 141 (40.5) | 67 (27.0) | 54 (28.4) | 17 (25.4) | 55 (39.6) | 35 (28.0) | 75 (45.7) | 23 (34.3) | 62 (33.9) | 38 (29.7) | 91 (38.1) | 58 (19.5) | 192 (25.9) | 37 (25.2) | 124 (30.5) |
| **KPS 100 (%)** | 199 (8.4) | 534 (10.5) | 19 (7.4) | 132 (17.6) | 31 (7.5) | 58 (10.6) | 35 (16.7) | 77 (12.1) | 18 (6.1) | 62 (8.4) | 7 (6.7) | 8 (2.3) | 40 (16.1) | 36 (18.9) | 2 (3.0) | 8 (5.8) | 13 (10.4) | 34 (20.7) | 7 (10.4) | 29 (15.8) | 10 (7.8) | 25 (10.5) | 8 (2.7) | 32 (4.3) | 9 (6.1) | 33 (8.1) |
| **Missing** | 66 (2.8) | 123 (2.4) | 0 (0.0) | 2 (0.3) | 20 (4.8) | 34 (6.2) | 4 (1.9) | 18 (2.8) | 1 (0.3) | 9 (1.2) | 4 (3.8) | 18 (5.2) | 23 (9.3) | 31 (16.3) | 9 (13.4) | 6 (4.3) | 1 (0.8) | 0 (0.0) | 0 (0.0) | 1 (0.5) | 3 (2.3) | 1 (0.4) | 1 (0.3) | 3 (0.4) | 0 (0.0) | 0 (0.0) |
| **ASA1** | 331 (14.0) | 984 (19.4) | 44 (17.1) | 161 (21.5) | 81 (19.5) | 161 (29.5) | 32 (15.3) | 147 (23.1) | 19 (6.4) | 66 (9.0) | 10 (9.6) | 72 (20.7) | 37 (14.9) | 30 (15.8) | 12 (17.9) | 49 (35.3) | 14 (11.2) | 36 (22.0) | 2 (3.0) | 14 (7.7) | 17 (13.3) | 34 (14.2) | 42 (14.1) | 141 (19.0) | 21 (14.3) | 73 (17.9) |
| **ASA2** | 1269 (53.7) | 2906 (57.2) | 144 (55.8) | 445 (59.4) | 211 (50.7) | 282 (51.6) | 82 (39.2) | 286 (44.9) | 176 (59.5) | 457 (62.0) | 59 (56.7) | 203 (58.3) | 123 (49.6) | 112 (58.9) | 29 (43.3) | 61 (43.9) | 61 (48.8) | 81 (49.4) | 44 (65.7) | 138 (75.4) | 72 (56.2) | 130 (54.4) | 179 (60.3) | 454 (61.2) | 89 (60.5) | 257 (63.1) |
| **ASA3** | 493 (20.9) | 774 (15.2) | 55 (21.3) | 114 (15.2) | 64 (15.4) | 48 (8.8) | 57 (27.3) | 113 (17.7) | 65 (22.0) | 112 (15.2) | 21 (20.2) | 47 (13.5) | 43 (17.3) | 23 (12.1) | 18 (26.9) | 18 (12.9) | 20 (16.0) | 19 (11.6) | 18 (26.9) | 26 (14.2) | 25 (19.5) | 52 (21.8) | 74 (24.9) | 135 (18.2) | 33 (22.4) | 67 (16.5) |
| **ASA4** | 21 (0.9) | 42 (0.8) | 0 (0.0) | 1 (0.1) | 5 (1.2) | 2 (0.4) | 2 (1.0) | 5 (0.8) | 4 (1.4) | 4 (0.5) | 2 (1.9) | 0 (0.0) | 1 (0.4) | 2 (1.1) | 0 (0.0) | 2 (1.4) | 1 (0.8) | 3 (1.8) | 3 (4.5) | 2 (1.1) | 1 (0.8) | 4 (1.7) | 1 (0.3) | 11 (1.5) | 1 (0.7) | 6 (1.5) |
| **ASA5** | 8 (0.3) | 16 (0.3) | 3 (1.2) | 4 (0.5) | 2 (0.5) | 1 (0.2) | 0 (0.0) | 0 (0.0) | 1 (0.3) | 2 (0.3) | 0 (0.0) | 0 (0.0) | 0 (0.0) | 0 (0.0) | 0 (0.0) | 4 (2.9) | 0 (0.0) | 2 (1.2) | 0 (0.0) | 0 (0.0) | 2 (1.6) | 3 (1.3) | 0 (0.0) | 0 (0.0) | 0 (0.0) | 0 (0.0) |
| **Missing** | 240 (10.2) | 359 (7.1) | 12 (4.7) | 24 (3.2) | 53 (12.7) | 52 (9.5) | 36 (17.2) | 86 (13.5) | 31 (10.5) | 96 (13.0) | 12 (11.5) | 26 (7.5) | 44 (17.7) | 23 (12.1) | 8 (11.9) | 5 (3.6) | 29 (23.2) | 23 (14.0) | 0 (0.0) | 3 (1.6) | 11 (8.6) | 16 (6.7) | 1 (0.3) | 1 (0.1) | 3 (2.0) | 4 (1.0) |
| **Chemo-radiotherapy** | 806 (34.1) | 3219 (63.4) | 92 (35.7) | 494 (66.0) | 183 (44.0) | 391 (71.6) | 75 (35.9) | 311 (48.8) | 100 (33.8) | 504 (68.4) | 34 (32.7) | 203 (58.3) | 51 (20.6) | 63 (33.2) | 13 (19.4) | 89 (64.0) | 28 (22.4) | 120 (73.2) | 28 (41.8) | 153 (83.6) | 48 (37.5) | 149 (62.3) | 94 (31.6) | 491 (66.2) | 60 (40.8) | 251 (61.7) |
| **Chemotherapy only** | 163 (6.9) | 256 (5.0) | 40 (15.5) | 72 (9.6) | 13 (3.1) | 21 (3.8) | 12 (5.7) | 35 (5.5) | 15 (5.1) | 16 (2.2) | 8 (7.7) | 15 (4.3) | 31 (12.5) | 17 (8.9) | 0 (0.0) | 2 (1.4) | 7 (5.6) | 5 (3.0) | 5 (7.5) | 5 (2.7) | 4 (3.1) | 10 (4.2) | 26 (8.8) | 48 (6.5) | 2 (1.4) | 10 (2.5) |
| **Radiotherapy only** | 342 (14.5) | 588 (11.6) | 30 (11.6) | 82 (10.9) | 48 (11.5) | 48 (8.8) | 19 (9.1) | 62 (9.7) | 48 (16.2) | 93 (12.6) | 8 (7.7) | 22 (6.3) | 32 (12.9) | 36 (18.9) | 12 (17.9) | 29 (20.9) | 31 (24.8) | 17 (10.4) | 2 (3.0) | 4 (2.2) | 20 (15.6) | 22 (9.2) | 54 (18.2) | 101 (13.6) | 38 (25.9) | 72 (17.7) |
| **None** | 905 (38.3) | 761 (15.0) | 94 (36.4) | 96 (12.8) | 172 (41.3) | 86 (15.8) | 69 (33.0) | 94 (14.8) | 127 (42.9) | 121 (16.4) | 41 (39.4) | 54 (15.5) | 50 (20.2) | 23 (12.1) | 41 (61.2) | 18 (12.9) | 58 (46.4) | 20 (12.2) | 31 (46.3) | 21 (11.5) | 53 (41.4) | 56 (23.4) | 123 (41.4) | 102 (13.7) | 46 (31.3) | 70 (17.2) |
| **Missing** | 146 (6.2) | 257 (5.1) | 2 (0.8) | 5 (0.7) | 0 (0.0) | 0 (0.0) | 34 (16.3) | 135 (21.2) | 6 (2.0) | 3 (0.4) | 13 (12.5) | 54 (15.5) | 84 (33.9) | 51 (26.8) | 1 (1.5) | 1 (0.7) | 1 (0.8) | 2 (1.2) | 1 (1.5) | 0 (0.0) | 3 (2.3) | 2 (0.8) | 0 (0.0) | 0 (0.0) | 1 (0.7) | 4 (1.0) |
